# The Major Histocompatibility Complex (MHC) in Schizophrenia: A Review

**DOI:** 10.4172/2155-9899.1000479

**Published:** 2016-12-21

**Authors:** Ryan Mokhtari, Herbert M Lachman

**Affiliations:** 1 Department of Psychiatry and Behavioral Sciences, Albert Einstein College of Medicine, 1300 Morris Park Ave., Bronx, New York, USA; 2 Department of Genetics, Albert Einstein College of Medicine, 1300 Morris Park Ave., Bronx, New York, USA; 3 Department of Neuroscience, Albert Einstein College of Medicine, 1300 Morris Park Ave., Bronx, New York, USA; 4 Department of Medicine, Albert Einstein College of Medicine, 1300 Morris Park Ave., Bronx, New York, USA

**Keywords:** Schizophrenia, MHC

## Abstract

Epidemiological studies and mouse models suggest that maternal immune activation, induced clinically through prenatal exposure to one of several infectious diseases, is a risk factor in the development of schizophrenia. This is supported by the strong genetic association established by genome wide association studies (GWAS) between the human leukocyte antigen (HLA) locus and schizophrenia. HLA proteins (also known in mice as the major histocompatibility complex; MHC) are mediators of the T-lymphocyte responses, and genetic variability is well-established as a risk factor for autoimmune diseases and susceptibility to infectious diseases. Taken together, the findings strongly suggest that schizophrenia risk in a subgroup of patients is caused by an infectious disease, and/or an autoimmune phenomenon. However, this view may be overly simplistic. First, MHC proteins have a non-immune effect on synaptogenesis by modulating synaptic pruning by microglia and other mechanisms, suggesting that genetic variability could be compromising this physiological process. Second, some GWAS signals in the HLA locus map near non-HLA genes, such as the histone gene cluster. On the other hand, recent GWAS data show association signals near B-lymphocyte enhancers, which lend support for an infectious disease etiology. Thus, although the genetic findings implicating the HLA locus are very robust, how genetic variability in this region leads to schizophrenia remains to be elucidated.

## Introduction

Emerging evidence suggests that the interactions between the immune system and the CNS are more complex than once thought. Despite their markedly different functions, these two systems share fundamental mechanisms at the molecular and cellular levels [1,2], implying a common evolutionary origin between them [3]. Recent findings have shown that the interplay between the immune and the nervous systems extends beyond the classical immune-mediated neuronal pathologies, such as multiple sclerosis, indicating immune dysregulation as a major factor in the etiopathogenesis of several psychiatric and neurodevelopmental disorders [4–6].

Schizophrenia has been extensively studied in relation to dysregulation of both innate and adaptive immune systems contributing to disease pathology [7–9]. Epidemiological studies revealed that schizophrenia is associated with a history of prenatal infection with agents such as *Toxoplasma gondii*, cytomegalovirus, and influenza and herpes viruses [10,11]. In addition, schizophrenia is associated with an increased risk of autoimmune diseases, such as type 1 diabetes, multiple sclerosis, and autoimmune hepatitis [12], and altered levels of inflammatory biomarkers, such as IL1β, IL6, IL10, and TNFα have been found [13–15].

Animal models have shown that maternal immune activation (MIA), initiated by infection or an autoimmune process, can increase the risk of neurodevelopmental disorders [16,17]. The “Cytokine Hypothesis” of schizophrenia posits that MIA leads to an increase in fetal brain cytokines, which alters neuronal connectivity, leading to schizophrenia-like behaviors [18,19].

However, one of the most fundamental levels of immune-CNS interaction emerges from the Human Leukocyte Antigen (HLA) locus (Major Histocompatibility Complex (MHC) in mice; HLA and MHC are used interchangeably); genome-wide association studies (GWAS) show that the HLA locus is one of the most significant determinants of schizophrenia susceptibility [20–22].

### MHC structure and functions

MHC comprises a family of surface proteins that regulate the adaptive immune system. The HLA locus on chromosome 6 (6p21.3–22.1) spans 7.6 Mb, comprising HLA classes I, II, III, as well as extended classes I and II sub-regions [23]. This region is highly polygenic and polymorphic [23,24]. In fact, the MHC family of genes is the most polymorphic known in vertebrates [25]. Class-I MHC molecules (MHCI) are found on the surface of all nucleated cells, where they present non-self protein fragments to cytotoxic T cells. MHCI molecules are heterodimers, comprised of a transmembrane α heavy chain, encoded by the polymorphic HLA locus, and a soluble beta2-microglobulin (β2m) light chain, encoded by the beta-2 microglobulin gene, which is not polymorphic; a third peptide component binds to the heavy chain [26].

The primary function of MHC involves processing and presentation of antigens, and the recognition of self-versus non-self antigens. However, many genes within the MHC locus code for proteins that function as transcription factors, receptors, ligands, and signaling molecules, playing major roles in non-immune processes as well [23]. Several non-immune functions have been proposed for MHC, including a possible role in animal mate choice and sexual selection [27]. Recently, there has been increasing evidence for the involvement of MHCI in fundamental functions of the CNS, including neurogenesis, neuronal differentiation and migration, and synaptic plasticity [28–31]. These core processes have all been implicated in the etiopathogenesis of schizophrenia [32–35].

### MHCI expression in the CNS

The brain used to be viewed as an "immune privileged" organ [36]. Similarly, healthy neurons were traditionally supposed to lack MHC molecules on their surface [37]. However, recent studies demonstrated the expression of MHCI proteins in mammalian brains [38]. The spatial and temporal distribution of MHCI expression is developmentally regulated, and the highest levels are expressed during the early postnatal development [30,39]. Later into adulthood, the levels of MHCI decline to a minimum [39,40], until they rise again with aging, albeit mainly in glial cells [41]. MHCI has been located on dendrites and axons, and at synapses, both pre- and postsynaptically [30,39].

Expression of MHCI in microglia has been suggested to contribute to their synaptic pruning function [42]. Recently, the "excessive pruning" hypothesis of schizophrenia was substantiated with the finding that schizophrenia risk is associated with variations in the complement component 4 (C4) genes, leading to increased C4 expression in microglia; in mice, increased C4 expression was found to increase in synapse elimination in the postnatal brain [43]. Neurons also express MHCI interacting partners, including components of their receptors, such as CD3ζ [44] and PirB [45].

While the neuronal expression of MHCI occurs in several regions of the CNS, including cerebral cortex, substantia nigra, olfactory bulb, brainstem, and spinal cord [39,46], the highest levels of expression have been reported in the hippocampus [28]. During normal development, however, levels of MHCI mRNA change dramatically in different regions, each of which shows a distinct mosaic pattern of neuronal expression, suggesting that MHCI may have diverse neuronal functions [47]. Postmortem studies on brains of schizophrenia patients versus controls showed that expression of MHCI may be upregulated or downregulated in different brain regions [48].

### Role of MHCI in CNS structural integrity

The structural integrity of the CNS is affected by MHCI, as evidenced by MHCI deficient mice showing enlarged ventricles [47], which is one of the frequently reported structural abnormalities in patients with schizophrenia [49]. In humans, highly significant associations were found between common variants in the MHC region and the cerebral ventricular size, specifically in schizophrenia patients [50]. Similarly, the volume and asymmetry of the human thalamus is related to genetic variation within the HLA region, with single nucleotide polymorphisms (SNPs) from a locus that previously associated with schizophrenia [51]. Asymmetry of the hippocampus has also been linked to MHCI, as β2m deficient mice did not show normal structural and functional asymmetries in hippocampal circuitry [52]. Abnormal hemispheric asymmetry, both in volume and in connectivity, has been reported in patients with schizophrenia [53–55]. Also, an aberrant hemispheric pattern of expression of HLA class II (HLA-DR) on microglia has been reported in schizophrenia [56].

## Role of MHCI in neurodevelopment

MHCI is expressed in during the earliest stages of the developing mammalian brain [40]. Expression of MHCI in neural progenitor cells and prenatal neurons occur earlier than the development of the adaptive immune system, suggesting non-immune roles of MHCI in brain development. The earliest stages of neuronal differentiation, i.e., neuronal polarization and neurite outgrowth, are regulated by MHCI in embryonic hippocampal neurons [57]. Soluble forms of MHCI (sMHCI) have been shown to negatively regulate neurite outgrowth in the embryonic mouse retina [58]. Interestingly, the neuronal inhibitory effect of self-MHCI is greater than that of non-self MHCI [59]. The non-classical MHCI molecule H2-Mv contributes to the structural organization of the mouse olfactory system [60]. In addition, MHCI co-receptors such as CD3ζ [61] and LY49 [62] have been implicated in the regulation of neuronal outgrowth and development.

### Role of MHCI in synaptic function and plasticity

Similar to the negative regulation of neurite outgrowth and dendritic branching, MHCI also negatively affects the establishment of neuronal connections and synapse density in the brain [30,39]. β2m deficient mice showed an increase in both glutamatergic and GABAergic synapse density, while overexpressing H2-Kb, a specific form of MHCI, decreased the density of both types of synapses [30]. This indicates that MHCI molecules influence the balance of excitatory-inhibitory neurons in the brain [30]. Since many cognitive symptoms of schizophrenia are thought to be related to an imbalance between inhibitory GABA and excitatory glutamate neurotransmission in the dorsolateral prefrontal cortex [63–66], it is conceivable that the cognitive deficits seen in schizophrenia could be due in part to dysregulation of MHCI expression or function adversely affecting GABAergic/glutamatergic balance.

MHCI has important roles in both activity-dependent (Hebbian) plasticity and also homeostatic plasticity (synaptic scaling) [28,31,67]. Activity-dependent plasticity is critical during periods of neurodevelopment when early experiences have long-term influences on neuronal connectivity and refinement: this process has been linked to MHCI [29,30]. This was first discovered when the blockade of neuronal activity in visual circuits reduced the expression of MHCI in the cat fetal brain [38]. Both forms of activity-dependent synaptic plasticity, long-term potentiation (LTP) and long-term depression (LTD), are affected in the nucleus accumbens of MHCI deficient mice [68], raising the possibility that MHCI may be involved in reward based learning. Mice with knockouts of the classical MHCI molecules, H2-Kb and H2-Db, showed a lower threshold for induction of LTD in the cerebellum, which suggests that MHCI has a role in synaptic plasticity and motor learning [69]. When the late persistent phase of long-term potentiation (L-LTP) is induced by an active form of cAMP Response Element Binding (CREB) protein in mouse hippocampal neurons, MHCI molecules were found among the most prominent differentially expressed genes [70]. Synaptic activity-induced LTP in the dentate gyrus of awake, freely moving rats was associated with altered expression of genes related to both MHC class I and class II [71]. Also, MHCI co-receptors, CD3ζ and PirB have been proposed to mediate the effects of MHCI on synaptic plasticity [45,72].

MHCI also mediates homeostatic plasticity, which is the network dependent adjustment of neuronal excitability [28,31]. Whole-cell recordings of hippocampal neurons from mouse knockouts of both β2m and TAP1 (a protein required for MHCI peptide transport) showed an increase in the frequency of miniature excitatory postsynaptic currents (mEPSCs), a measure of synaptic strength [28]. Correspondingly, MHCI overexpression decreased mEPSC frequency [30]. Synaptic disconnection has been suggested as a core pathological feature in schizophrenia [32,35]. The density of dendritic spine synapses was decreased in pyramidal neurons of individuals with schizophrenia [73]. An important effect of MHCI on synaptic plasticity, in both Hebbian and homeostatic forms, is the modulation of glutamate receptors, NMDAR and AMPAR [74]. MHCI-deficient hippocampal synapses showed a decreased AMPA/NMDA ratio, with downstream effects on AMPA receptor trafficking [74].

**Figure 1:**
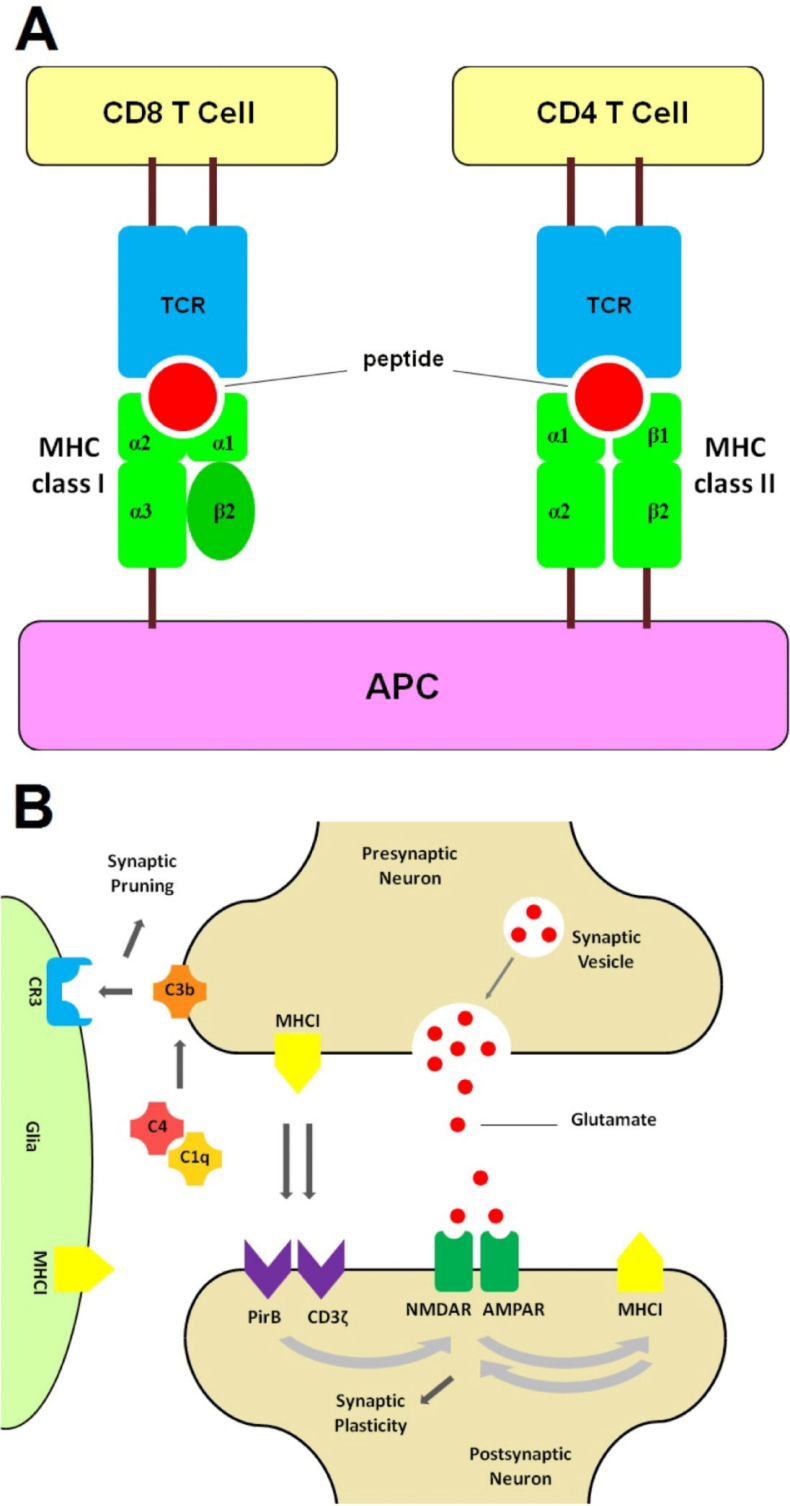
**A)** The structures of MHC class-I and class-II molecules and their binding to T lymphocytes. **B)** Possible mechanisms by which MHCI exerts its effects on synapses. See text for details: TCR: T-cell Receptor; APC: Antigen Presenting Cell; C1q, C4, C3b, C3R: Complement components.

According to the "glutamate hypothesis of schizophrenia", hypofunction of glutamatergic signaling via NMDAR is the pivotal mechanism of schizophrenia pathology [75]. NMDAR hypofunction may be associated with early-life oxidative stress and immune reactivity [76]. More specifically, in the case of anti-NMDAR encephalitis, autoantibodies against the NR1 subunit of the NMDAR contribute to the hypofunction of these receptors, which is the likely cause of the psychosis and other cognitive deficits seen in this disease [77]. These findings suggest that MHCI inhibition of NMDAR function may be a crucial step in the pathogenesis of schizophrenia (Figure 1).

Despite the accumulating evidence for the involvement of MHCI in core neurodevelopmental processes, the mechanisms by which MHCI molecules exert their effects still needs to be elucidated. Likewise, the connection between the neurodevelopmental functions of MHCI and the pathogenesis of schizophrenia remains to be established.

### Genomic association of HLA variants with schizophrenia

The first evidence for association between the HLA locus and schizophrenia risk dates back to a study published more than four decades ago [78]. Since then, many genetic linkage studies have been conducted in different ethnic populations, and several HLA alleles (e.g. HLA-A9, HLA-A10, HLA-DRB1, HLA-DQB1) were found to be linked to schizophrenia [79,80]. However, these studies were not consistently replicated. Also, many of the initial positive results may have been due to type I error caused by small sample sizes and other limitations affecting linkage studies of genetically complex disorders [79,81].

GWAS introduced a new paradigm for analyzing common complex traits, such as schizophrenia [82]. Analyzing more than a million SNPs in large sample sizes dramatically improved the statistical validity of the associations. A breakthrough in the field of neuroimmunology occurred in 2009, when three simultaneously published GWAS reported that several common variants within the MHC locus are strongly associated with the risk of schizophrenia [20–22]. A meta-analysis of these studies (8,008 cases and 19,077 controls) combined the p-values of all SNPs from the most significant regions of each study, and found seven significant SNPs in a region of strong linkage disequilibrium (r^2^>0.9) on chromosome 6p22.1 [21]. The strongest association (p=9 × 10^−9^) was in rs13194053, which is near a cluster of histone genes (including *HIST1H2BJ* and *HIST1H2AH*) and several immune-related genes. This finding indicates that chromatin modification, transcriptional regulation and autoimmunity/infection are among the likely processes involved in the etiopathogenesis of schizophrenia [21]. The most significant SNP in the International Schizophrenia Consortium study was rs3130375, which is in linkage with the *RPP21* gene, which encodes a ribonuclease involved in tRNA processing [20].

Although the first group of GWAS was performed only on individuals of European ancestry, subsequent GWAS on other ethnic populations consistently replicated the association of schizophrenia risk with many of the previously identified MHC variants, and some new HLA variants in Asian populations reached genome wide significance as well [83,84]. Subsequent studies, meta-analyses, pathway analyses, and expression quantitative trait loci (eQTL) analyses, further substantiated the association of schizophrenia with the HLA locus [85–93]. The largest combined analysis of GWAS samples (20,476 cases and 36,737 control subjects) identified highly significant SNPs such as rs2021722 (p=1.05 × 10^−14^) in schizophrenia [91]. However, the high level of linkage disequilibrium in the MHCI region makes it difficult to find the actual disease-associated variants. Consistent replication of HLA association in diverse populations and in different meta-analyses underscores the validity and reliability of this finding, reassuring that the results are not due to the population stratification artifacts, a common cause of type I error. HLA locus association is now considered the most significant and consistent finding in schizophrenia GWAS [94].

Among the top SNPs in the HLA locus consistently replicated in schizophrenia GWAS, some are in close proximity to genes previously associated with schizophrenia susceptibility. Two different significant SNPs (rs3131296 and rs2071287) map closely to the gene *NOTCH4* (neurogenic locus notch homolog 4), which codes for a transmembrane protein critical for neurodevelopmental processes; *NOTCH4* has been implicated as a schizophrenia risk gene in other studies [95,96]. Two additional SNPs in *NOTCH4* (rs3132935 and rs3132947) were also found to be significant in a family-based replication of a GWAS meta-analysis [90]. *NOTCH4* is a non-HLA gene that maps to the HLA locus, suggesting that that both immune and non-immune functions of genes in the HLA locus may be involved in the pathogenesis of schizophrenia. Moreover, *NOTCH4* has been associated with some cognitive endophenotypes of schizophrenia [97], and also with some frontal lobe structural variations between patients and controls [98].

In an extensive meta-analysis [86], the most significant SNP was rs2021722, which is located within the *HLA* locus, in *TRIM26* (Tripartite Motif Containing 26). This gene codes for a protein with unknown function, but it has been linked to immune-related pathways [99]. In an expression QTL analysis of the top SNPs from the aforementioned meta-analysis, only two genes (*TRIM26* and *HLA-DRB3*) showed differential expression between schizophrenia patients and controls, with the same direction of change expected from the meta-analysis [88]. Additional SNPs were also found in linkage disequilibrium with *TRIM26* in an independent GWAS and meta-analysis [100].

## Conclusion

Although some challenges to the robustness of association of MHC locus with schizophrenia (e.g. population stratification bias) have been addressed by advanced biotechnological and bioinformatics methods, research in this area still faces important challenges concerning efforts to understand the exact neurobiological mechanisms by which HLA variants contribute to the risk of schizophrenia. Despite the overall replication consistency for the entire HLA locus, individual significant SNPs within the region have not been consistently replicated in different GWAS and meta-analyses, even when the replication is attempted within the same ethnic group. Similarly, the location of the best association signals differs between the GWA studies [101]. This may be due to the genetic heterogeneity of schizophrenia, not only among different ethnic populations, but also at the individual level.

Another challenge is related to the localization of SNP signals to plausible risk genes, finding meaningful molecular pathways, and functionally validation them. Similarly, the significant SNPs that do not map to exons cannot be easily assigned to particular functions that could potentially explain the risk of schizophrenia [81].

Finally, the immune system dysregulations in schizophrenia should not be reduced to the role of the HLA locus. For example, in a large multi-stage schizophrenia GWAS [94], association signals were found at enhancers that are active in B-lymphocyte lineages involved in acquired immunity (CD19 and CD20 positive cells), which remained significant even after excluding the extended HLA locus. This finding indicates that the immune system involvement in schizophrenia pathogenesis extends beyond the effects of the HLA locus. In addition, while the non-immune effects of MHC on synaptogenesis are intriguing with respect to schizophrenia pathogenesis, the association to B-lymphocyte enhancers suggests that infectious disease and/or autoimmune phenomena are still plausible pathways towards explaining the positive association between the HLA gene locus and schizophrenia.
